# Deep Learning Techniques to Diagnose Lung Cancer

**DOI:** 10.3390/cancers14225569

**Published:** 2022-11-13

**Authors:** Lulu Wang

**Affiliations:** Biomedical Device Innovation Center, Shenzhen Technology University, Shenzhen 518118, China; lwang381@hotmail.com or wanglulu@sztu.edu.cn

**Keywords:** lung cancer, medical images, segmentation, classification, deep learning, convolutional neural network

## Abstract

**Simple Summary:**

This study investigates the latest achievements, challenges, and future research directions of deep learning techniques for lung cancer and pulmonary nodule detection. Hopefully, these research findings will help scientists, investigators, and clinicians develop new and effective medical imaging tools to improve lung nodule diagnosis accuracy, sensitivity, and specificity.

**Abstract:**

Medical imaging tools are essential in early-stage lung cancer diagnostics and the monitoring of lung cancer during treatment. Various medical imaging modalities, such as chest X-ray, magnetic resonance imaging, positron emission tomography, computed tomography, and molecular imaging techniques, have been extensively studied for lung cancer detection. These techniques have some limitations, including not classifying cancer images automatically, which is unsuitable for patients with other pathologies. It is urgently necessary to develop a sensitive and accurate approach to the early diagnosis of lung cancer. Deep learning is one of the fastest-growing topics in medical imaging, with rapidly emerging applications spanning medical image-based and textural data modalities. With the help of deep learning-based medical imaging tools, clinicians can detect and classify lung nodules more accurately and quickly. This paper presents the recent development of deep learning-based imaging techniques for early lung cancer detection.

## 1. Introduction

Lung cancer is the most frequent cancer and the cause of cancer death, with the highest morbidity and mortality in the United States [1]. In 2018, GLOBOCAN estimated approximately 2.09 million new cases and 1.76 million lung cancer-related deaths [2]. Lung cancer cases and deaths have increased significantly globally [2]. Approximately 85–88% of lung cancer cases are non-small cell lung carcinoma (NSCLS), and about 12–15% of lung cancer cases are small cell lung cancer (SCLC) [3]. Early lung cancer diagnosis and intervention are crucial to increase the overall 5-year survival rate due to the invasiveness and heterogeneity of lung cancer [4].

Over the past two decades, various medical imaging techniques, such as chest X-ray, positron emission tomography (PET), magnetic resonance imaging (MRI), computed tomography (CT), low-dose CT (LDCT), and chest radiograph (CRG), have been extensively investigated for lung nodule detection. Although CT is the golden standard imaging tool for lung nodule detection, it can only detect apparent lung cancer with high false-positive rates and produces harmful X-ray radiation [5]. LDCT has been proposed to reduce harmful radiation to detect lung cancer [6]. However, cancer-related deaths were concentrated in subjects undergoing LDCT. 2-deoxy-18F-fluorodeoxyglucose (18F-FDG) PET has been developed to improve the detection performance of lung cancer [7]. 18F-FDG PET produces semi-quantitative parameters of tumor glucose metabolism, which is helpful in the diagnosis of NSCLC [8]. However, 18F-FDG PET requires further evaluation of patients with NSCLC. Some new imaging techniques, such as magnetic induction tomography (MIT), have been developed for early-stage cancer cell detection [9]. However, this technique lacks clinical validation of human subjects. 

Many computer-aided detection (CAD) systems have been extensively studied for lung cancer detection and classification [10,11]. Compared to trained radiologists, CAD systems provide better lung nodules and cancer detection performance in medical images. Generally, the CAD-based lung cancer detection system includes four steps: image processing, extraction of the region of interest (ROI), feature selection, and classification. Among these steps, feature selection and classification play the most critical roles in improving the accuracy and sensitivity of the CAD system, which relies on image processing to capture reliable features. However, benign and malignant nodule classification is a challenge. Many investigators have applied deep learning techniques to help radiologists make more accurate diagnoses [12,13,14,15]. Previous studies have confirmed that deep learning-based CAD systems can effectively improve the efficiency and accuracy of medical diagnosis, especially for diagnosing various common cancers, such as lung and breast cancers [16,17]. Deep learning-based CAD systems can automatically extract high-level features from original images using different network structures than traditional CAD systems. However, deep learning-based CAD systems have some limitations, such as low sensitivity, high FP, and time consumption. Therefore, a rapid, cost-effective, and highly sensitive deep learning-based CAD system for lung cancer prediction is urgently needed.

The deep learning-based lung imaging techniques research mainly includes pulmonary nodule detection, segmentation, and classification of benign and malignant pulmonary nodules. Researchers mainly focus on developing new network structures and loss functions to improve the performance of deep learning models. Several research groups have recently published review papers on deep learning techniques [18,19,20]. However, deep learning techniques have developed rapidly, and many new methods and applications have emerged every year. This research has appeared with content that previous studies cannot cover.

This paper presents recent achievements in lung cancer segmentation, detection, and classification using deep learning methods. This study highlights current state-of-the-art deep learning-based lung cancer detection methods. This paper also highlights recent achievements, relevant research challenges, and future research directions. The rest of the paper is structured as follows. Section 2 describes the currently available medical lung imaging techniques for lung cancer detection; Section 3 reviews some recently developed deep learning-based imaging techniques; Section 4 presents lung cancer prediction using deep learning techniques; Section 5 describes the current challenges and future research directions of deep learning-based lung imaging methods; and Section 6 concludes this study.

## 2. Lung Imaging Techniques 

Medical imaging tools help radiologists diagnose lung disease. Among these medical imaging approaches, CT offers more advantages, including size, location, characterization, and lesion growth, which could identify lung cancer and nodule information. 4D CT provides more precise targeting of the administered radiation, which significantly impacts lung cancer management [21]. Lakshmanaprabu et al. [22] developed an automatic detection system based on linear discriminate analysis (LDA) and an optimal deep neural network (ODNN) to classify lung cancer in CT lung images. The LDA reduced the extracted image features to minimize the feature dimension. The ODNN was applied and optimized by a modified gravitational search algorithm to provide a more accurate classification result. Compared to CT, LDCT is more sensitive to early-stage lung nodules and cancer detection with reduced radiation. However, it does not help reduce lung cancer mortality. It is recommended that LDCT be carried out annually for high-risk smokers aged 55 to 74 [23].

PET produces much higher sensitivity and specificity for lung nodule detection than CT due to reactive or granulomatous nodal disease [24]. PET offers a good correlation with longer progression times and overall survival rates. 18F-FDG PET has been applied to diagnose solitary pulmonary nodules [25]. 18F-FDG PET is a crucial in-patient selection and advanced NSCLC for radical radiotherapy. PET-assisted radiotherapy offers more accuracy [26] and manages about 32% of patients with stage IIIA lung cancer [27]. 18F-FDG PET provides a significant response assessment in patients with NSCLC undergoing induction chemotherapy.

MRI is the most potent lung imaging tool without ionizing radiation, but it provides insufficient information with high costs and time-consuming limitations. It failed to detect about 10% of small lung nodules (4–8 mm in diameter) [28]. MRI with ultra-short echo time (UTE) can improve signal intensity and reduce lung susceptibility artifacts. MRI with UTE is sensitive for detecting small lung nodules (4–8 mm) [29]. MRI achieves a higher lung nodule detection rate than LDCT. MRI with different pulse sequences also improved lung nodule detection sensitivity. The authors investigated T1-weighted and T2-weighted MRI to detect small lung nodules [30,31]. Compared to 3T 1.5 MRI, 1.5T MRI is much easier to identify ground glass opacities [32]. Ground glass opacities were successfully detected in 75% of subjects with lung fibrosis who received 1.5T MRI with SSFP sequences [33]. MRI with T2-weighted fast spin echo provides similar or even better performance for ground glass infiltrate detection in immunocompromised subjects [34].

Several research groups have recently investigated the feasibility of using MIT for lung disease detection [35,36]. However, due to the lack of measurement systems, expensive computational electromagnetic models, low image resolution, and some other challenges, MIT technology still has a long way to go before it can be widely used as a commercial imaging tool in clinical conditions.

**Figure 1:**
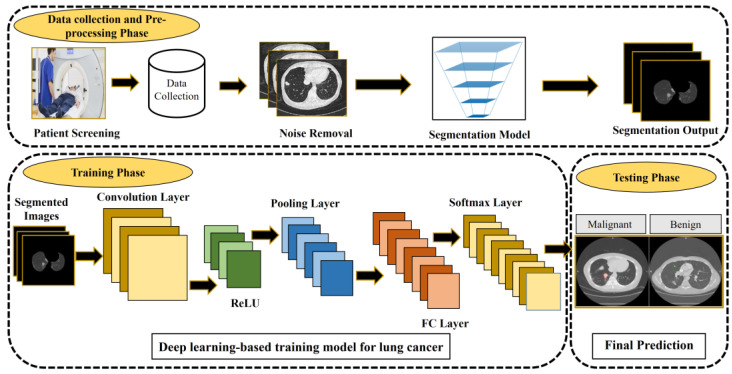
CAD-based lung cancer detection system [14]. The figure is reused from reference [14]; no special permission is required to reuse all or part of articles published by MDPI, including figures and tables. For articles published under an open-access Creative Common CC BY license.

Medical imaging approaches play an essential strategy in early-stage lung cancer detection and improve the survival rate. However, these techniques have some limitations, including high false positives, and cannot detect lesions automatically. Several CAD systems have been developed for lung cancer detection [37,38]. As shown in Figure 1, a CAD-based lung nodule detection system [14] usually consists of three main phases: data collection and pre-processing, training, and testing. There are two types of CAD systems: the detection system identifies specific anomalies according to interest regions, and the diagnostic system analyses lesion information, such as type, severity, stage, and progression. 

## 3. Deep Learning-Based Imaging Techniques 

A deep learning-based CAD system has been reported as a promising tool for the automatic diagnosis of lung disease in medical imaging with significant accuracy [34,35,36]. The deep learning model is a neural network model with multiple levels of data representation. The deep learning approaches can be grouped into unsupervised, reinforcement, and supervised learning.

Unsupervised learning does not require user guidance, which analyzes the data and then sorts inherent similarities between the input data. Therefore, semi-supervised learning is a mixed model that can provide a win-win situation, even with different challenges. Semi-supervised learning techniques use both labeled and unlabeled data. With the help of labeled and unlabeled data, the accuracy of the decision boundary becomes much higher. Auto-Encoders (AE), Restricted Boltzmann Machines (RBM), and Generative Adversarial Networks (GAN) are good at clustering and nonlinear dimensionality reduction. A large amount of labeled data is usually required during training, which increases cost, time, and difficulty. Researchers have applied deep clustering to reduce labeling and make a more robust model [39,40]. 

Convolutional neural networks (CNN), deep convolutional neural networks (DCNN), and recurrent neural networks (RNN) are the most widely used unsupervised learning algorithms in medical images. CNN architecture is one of the most widely used supervised deep learning approaches for lesion segmentation and classification because less pre-processing is required. CNN architectures have recently been applied to medical images for image segmentation (such as Mask R-CNN [41]) and classification (such as AlexNet [42] and VGGNet [43]). DCNN architectures usually contain more layers with complex nonlinear relationships, which have been used for classification and regression with reasonable accuracy [44,45,46]. RNN architecture is a higher-order neural network that can accommodate the network output to re-input [47]. RNN applies the Elman network with feedback links from the hidden layer to the input layer, which has the potential to capture and exploit cross-slice variations to incorporate volumetric patterns of nodules. However, RNN has a vanishing gradient problem. 

The reinforcement learning technique was first applied in Google Deep Mind in 2013 [48]. Since then, reinforcement learning approaches have been extensively investigated to improve lung cancer detection accuracy, sensitivity, and specificity. Semi-supervised learning approaches, such as deep reinforcement learning and generative adversarial networks, use labeled datasets. 

Supervised learning usually involves a learning algorithm, and labels are assigned to the input data according to the labeling data during training. Various supervised deep learning approaches have been applied to CT images to identify abnormalities with anatomical localization. These approaches have some drawbacks, such as the large amount of labeled data required during training, the assumption of fixed network weights upon training completion, and the inability to be improved after training. Thus, a few-shot learning (FSL) model is developed to reduce data requirements during training. 

## 4. Lung Cancer Prediction Using Deep Learning

This section presents recent achievements in lung cancer and nodule prediction using deep learning techniques. The processing includes image pre-processing, lung nodule segmentation, detection, and classification. 

### 4.1. Imaging Pre-Processing Techniques and Evaluation

#### 4.1.1. Pre-Processing Techniques

The pre-processed images are injected into a deep learning algorithm with specific architecture and training and tested on the image datasets. The image noise affects the precision of the final classifier. Several noise reduction approaches, such as median filter [48], Wiener filter [49], and non-local means filter [50], have been developed for pre-processing to improve accuracy and generalization performance. After denoising, a normalization method, such as min-max normalization, is required to rescale the images and reduce the complexity of image datasets.

#### 4.1.2. Performance Metrics

Several performance metrics have been used to evaluate the performance of deep learning algorithms, including accuracy, precision, sensitivity, specificity, F1_score, error, mean squared error (MSE), receiver operation characteristic (ROC) curve, over-segmentation rate (OR), under-segmentation rate (UR), Dice similarity coefficient (DSC), Jaccard Score (JS), average symmetric surface distance (ASD), modified Hausdorff distance (MHD), and intersection over union (IoU). 

Accuracy assesses the capability concerning the results with the existing information features. Sensitivity is helpful for evaluation when FN is high. Precision is an effective measurement index when FP is high. The F1_score is applied when the class distribution is uneven. ROC can tune detection sensitivity. The area under the receiver operating characteristic curve (AUC) has been used to evaluate the proposed deep learning model. Larger values of accuracy, precision, sensitivity, specificity, AUC, DSC, and JS, and smaller values of Error, UR, OR, and MHD indicate better performance of a deep learning-based algorithm.

These performance metrics can be computed using the following equations [51,52]:

(1) $$\mathrm{Accuracy}=\frac{\mathrm{TP}+\mathrm{TN}}{\mathrm{TP}+\mathrm{TN}+\mathrm{FP}+\mathrm{FN}}$$

(2) $$\mathrm{Sensitivity}=\frac{\mathrm{TP}}{\mathrm{TP}+\mathrm{FN}}$$

(3) $$\mathrm{Specificity}=\frac{\mathrm{TN}}{\mathrm{TN}+\mathrm{FP}}$$

(4) $$\mathrm{Precision}=\frac{\mathrm{TP}}{\mathrm{TP}+\mathrm{FP}}$$

(5) $$F1\_\mathrm{score}=\frac{2\mathrm{TP}}{2\mathrm{TP}+\mathrm{FP}+\mathrm{FN}}$$

(6) $$\mathrm{Error}=\frac{\mathrm{FP}+\mathrm{FN}}{\mathrm{TP}+\mathrm{TN}+\mathrm{FP}+\mathrm{FN}}$$

(7) $$\mathrm{DSC}=\frac{2\mathrm{TP}}{2\mathrm{TP}+\mathrm{FP}+\mathrm{FN}}$$

(8) $$\mathrm{JS}=\frac{\mathrm{DSC}}{2-\mathrm{DSC}}$$

(9) $$\mathrm{MHD}\left(A,B\right)=\frac{1}{N_{a}}\overset{}{\underset{a\in A}{\sum }}\underset{b\in B}{\min}||a-b||$$

(10) $$\mathrm{IoU}=\frac{\mathrm{TP}}{\mathrm{TP}+\mathrm{FP}+\mathrm{TN}}$$

where TP (true positive) denotes the number of correct positives; TN (true negative) indicates the number of correct negatives; FP (false positive) means the number of incorrect positives; FN (false negative) denotes the number of incorrect negatives; B is the target object region, A denotes ground truth dataset, and $N_{a}$ is the number of pixels in A; IoU refers to the percentage of the intersection to the union of the ground truth and predicted areas and is a metric for various object detection and semantic segmentation problems.

### 4.2. Datasets

Lung image datasets play an essential role in evaluating the performance of deep learning-based algorithms for lung nodule classification and detection. Table 1 shows publicly available lung images and clinical datasets for assessing nodule classification and detection performance. 

### 4.3. Lung Image Segmentation

Image segmentation aims to recognize the voxel information and external contour of the region of interest. In medical imaging, segmentation is mainly used to segment organs or lesions to quantitatively analyze relevant clinical parameters and provide further guidance for follow-up diagnosis and treatment. For example, target delineation is crucial for surgical image navigation and tumor radiotherapy guidance.

Lung segmentation plays a crucial role in medical images for lesion detection, including thorax extraction (removes artifacts) and lung extraction (identifies the left and right lungs). Several threshold techniques, such as the threshold method [69], iterative threshold [70], Otsu threshold [71], and adaptive threshold [72,73], have been investigated for lung segmentation. Few research groups have investigated segmentation methods based on region and 3D region growth [74,75]. Kass et al. [76] first introduced the active contour model, and Lan et al. [77] applied the active contour model for lung segmentation. These techniques are manual segmentation and have many disadvantages, such as being relatively slow, prone to human error, scarcity of ground truth, and class imbalance. 

Several deep learning approaches have been investigated for lung segmentation. Wang et al. [78] developed a multi-view CNN (MV-CNN) for lung nodule segmentation, with an average DSC of 77.67% and an average ASD of 0.24 for the LIDC-IDRI dataset. Unlike conventional CNN, MV-CNN integrates multiple input images for lung nodule identification. However, it is difficult for MV-CNN to process 3D CT scans. Thus, a 3D CNN was developed to process volumetric patterns of cancerous nodules [79]. Sun et al. [80] designed a two-stage CAD system to segment lung nodules and FP reduction automatically. The first stage aims to identify and segment the nodules, and the second stage aims to reduce FP. The system was tested on the LIDC-IDRI dataset and evaluated by four experienced radiologists. The system obtained an average F1_score of 0.8501 for lung nodule segmentation. 

In 2020, Cao et al. [81] developed a dual-branch residual network (DB-ResNet) that simultaneously captures the multi-view and multi-scale features of nodules. The proposed DB-ResNet was evaluated on the LIDC-IDRI dataset and achieved a DSC of 82.74%. Compared to trained radiologists, DB-ResNet provides a higher DSC.

In 2021, Banu et al. [82] proposed an attention-aware weight excitation U-Net (AWEU-Net) architecture in CT images for lung nodule segmentation. The architecture contains two stages: lung nodule detection based on fine-tuned Faster R-CNN and lung nodule segmentation based on the U-Net with position attention-aware weight excitation (PAWE) and channel attention-aware weight excitation (CAWE). The AWEU-Net obtained DSC of 89.79% and 90.35%, IoU of 82.34%, and 83.21% for the LUNA16 and LIDC-IDRI datasets, respectively. 

Dutta [83] developed a dense recurrent residual CNN (Dense R2Unet) based on the U-Net and dense interconnections. The proposed method was tested on a lung segmentation dataset, and the results showed that the Dense R2UNet offers better segmentation performance than U-Net and ResUNet. 

Table 2 shows the recently developed lung nodule segmentation techniques. Among these approaches, SVM systems obtained an accuracy range of 92.6–98.1%, CNN-based systems obtained a specificity range of 77.67–91%, ResNet models obtained a DSC range of 82.74–98.1%, and U-Net segmentation systems achieved an accuracy range of 82.2–99.27%, precision range of 46.61–98.2%, recall range of 21.43–96.33%, and F1_score range of 24.64–99.1%, respectively. The DenseNet201 system obtained an accuracy of 97%, a sensitivity of 96.2%, a specificity of 97.5%, an AUC of 0.968, and an F1_score of 96.1%. Several segmentation methods, including SVM, Dense R2UNet, 3D Attention U-Net, Dense R2UNet, Res BCDU-Net, U-Net FSL, U-Net CT, U-Net PET, U-Net PET/CT, CNN, and DenseNet201, achieved high accuracy results (over 94%).

### 4.4. Lung Nodule Detection

Lung nodule detection is challenging because its shape, texture, and size vary greatly, and some non-nodules, such as blood vessels and fibrosis, have a similar appearance to lung nodules that often appear in the lungs. The processing includes two main steps: lung nodule detection and false-positive nodule reduction. Over the past few decades, researchers worldwide have extensively investigated machine learning and deep learning-based approaches for lung nodule detection. Chang et al. [106] applied the support vector machine (SVM) for nodules classification in ultrasound images. Nithila et al. [107] developed a lung nodule detection model based on heuristic search and particle clustering algorithms for network optimization. In 2005, Zhang et al. [108] developed a discrete-time cellular neural network (DTCNN) to detect small (2–10 mm) juxtapleural and non-pleural nodules in CT images. The method obtained a sensitivity of 81.25% at 8.29 FPs per scan for juxtapleural nodule detection and a sensitivity of 83.9% at 3.47 FPs per scan for non-pleural nodule detection. 

Hwang et al. [109] investigated the relationship between CT and commercial CAD to detect lung nodules. They also studied LDCT images with three reconstruction kernels (B, C, and L) from 36 human subjects. The sensitivities of 82%, 88%, and 82% for the nodules of B, C, and L were obtained for all images. Experimental results showed that CAD sensitivity could be elevated by combining data from 2 different kernels without radiation exposure. Young et al. [110] studied the effects on the performance of a CAD-based nodule detection model by reducing the CT dose. The CAD system was evaluated on the NLST dataset and obtained sensitivities of 35%, 20%, and 42.5% at the initial dose, 50% dose, and 25% dose, respectively. Tajbakhsh et al. [111] studied massive training ANN (MTANN) and CNN for lung nodule detection and classification. MTANN and CNN obtained AUCs of 0.8806 and 0.7755, respectively. MTANN performs better than CNN for lung nodule detection and classification.

Liu et al. [112] developed a cascade CNN for lung nodule detection. The transfer learning model was applied to train the network to detect nodules, and a non-nodule filter was introduced to the detection network to reduce false positives (FP). The proposed architecture effectively reduces FP in the lung nodule detection system. Li et al. [65] developed a lung nodule detection method based on a faster R-CNN network and an FP reduction model in thoracic MR images. In this study, a faster R-CNN was developed to detect lung nodules, and an FP reduction model was developed to reduce FP. The method was tested on the FAHGMU dataset and obtained a sensitivity of 85.2%, with 3.47 FP per scan. Cao et al. [113] developed a two-stage CNN (TSCNN) model for lung nodule detection. In the first stage, a U-Net based on ResDense was applied to detect lung nodules. A 3D CNN-based ensemble learning architecture was proposed in the second stage to reduce false-positive nodules. The proposed model was compared with three existing models, including 3DDP-DenseNet, 3DDP-SeResNet, and 3DMBInceptionNet.

Several 3D CNN models have been developed for lung nodule detection [114,115,116]. Perez et al. [117] developed a 3D CNN to automatically detect lung cancer and tested the model on the LIDC-IDRI dataset. The experimental results showed that the proposed method provides a recall of 99.6% and an AUC of 0.913. Vipparla et al. [118] proposed a multi-patched 3D CNN with a hybrid fusion architecture for lung nodule detection with reduced FP. The method was tested on the LUNA16 dataset and achieved a competition performance metric (CPM) of 0.931. Dutande et al. [119] developed a 2D–3D cascaded CNN architecture and compared it with existing lung nodule detection and segmentation methods. The results showed that the 2D–3D cascaded CNN architecture obtained a DCM of 0.80 for nodule segmentation and a sensitivity of 90.01% for nodule detection. Luo et al. [120] developed a 3D sphere representation-based center-point matching detection network (SCPM-Net) consisting of sphere representation and center-point matching components. The SCPM-Net was tested on the LUNA16 dataset and achieved an average sensitivity of 89.2% at 7 FPs per image for lung nodule detection. Franck et al. [121] investigated the effects on the performance of deep learning image reconstruction (DLIR) techniques on lung nodule detection in chest CT images. In this study, up to 6 artificial nodules were located within the lung phantom. Images were generated using 50% ASIR-V and DLIR with low (DL-L), medium (DL-M), and high (DL-H) strengths. No statistically significant difference was obtained between these methods (*p* = 0.987, average AUC: 0.555, 0.561, 0.557, and 0.558 for ASIR-V, DL-L, DL-M, and DL-H).

Table 3 shows recently developed lung nodule detection approaches using deep learning techniques. Among these approaches, the co-learning feature fusion CNN obtained the best accuracy of 99.29%, which is higher than other lung nodule detection approaches. Several networks, including 3D Faster R-CNN with U-Net-like encoder, YOLOv2, YOLOv3, VGG-16, DTCNN-ELM, U-Net++, MIXCAPS, and ProCAN, obtained good accuracy (>90%) of lung nodule detection.

### 4.5. Lung Nodule Classification

In recent years, investigators have studied various deep learning techniques to improve the performance of lung nodule classification [160,161,162,163,164,165,166,167,168,169,170,171,172,173]. The sensitivity and specificity of the SIFT-based classifier and SVM in the classification of pulmonary nodules reached 86% and 97% [160], 91.38%, and 89.56% [163], respectively. The accuracy, sensitivity, and specificity of multi-scale CNN and multi-crop CNN in lung nodule classification were 90.63%, 92.30%, and 89.47% [164], respectively, and 87%, 77%, and 93% [170], respectively. The accuracy of deep-level semantic networks and multi-scale CNN in lung nodule classification were 84.2% [167] and 86.84% [168], respectively. The CAD system developed by Cheng et al. [169] achieved the best accuracy of 95.6%, sensitivity of 92.4%, and specificity of 98.9% in the classification of pulmonary nodules. 

The comparative study results showed that the sensitivity and specificity of CNN and DBN for pulmonary nodule classification are 73.40% and 73.30%, 82.20%, and 78.70%, respectively [165]. Another comparative study showed that the sensitivity and specificity of CNN and ResNet in the classification of nodules are 76.64% and 89.50%, 81.97%, and 89.38%, respectively [171]. The combined application of CNN and RNN achieved accuracy, sensitivity, and specificity of 94.78%, 94.66%, and 95.14%, respectively, in classifying pulmonary nodules [172]. 

In 2019, Zhang et al. [174] used an ensemble learner of multiple deep CNN in CT images and obtained a classification accuracy of 84% for the LIDC-IDRI dataset. The proposed classifier achieved better performance than other algorithms, such as SVM, multi-layer perceptron, and random forests. 

Sahu et al. [175] proposed a lightweight multi-section CNN with a classification accuracy of 93.18% for the LIDC-IDRI dataset to improve accuracy. The proposed architecture could be applied to select the representative cross sections determining malignancy that facilitate the interpretation of the results. 

Ali et al. [176] developed a system based on transferable texture CNN that consists of nine layers to extract features automatically and classify lung nodules. The proposed method achieved an accuracy of 96.69% ± 0.72%, with an error of 3.30% ± 0.72% and a recall of 97.19% ± 0.57%, respectively.

Marques et al. [177] developed a multi-task CNN to classify malignancy nodules with an AUC of 0.783. Thamilarasi et al. [178] proposed an automatic lung nodule classifier based on CNN with an accuracy of 86.67% for the JSRT dataset. Kawathekar et al. [179] developed a lung nodule classifier using a machine-learning technique with an accuracy of 94% and an F1_score of 92% for the LNDb dataset. 

More recently, Radford et al. [180] proposed deep convolution GAN (DCGAN), Chuquicusma et al. [181] applied DCGAN to generate realistic lung nodules, and Zhao et al. [182] applied Forward and Backward GAN (F&BGAN) to classify lung nodules. The F&BGAN was evaluated on the LIDC-IDRI dataset and obtained the best accuracy of 95.24%, a sensitivity of 98.67%, a specificity of 92.47%, and an AUC of 0.98.

Table 4 shows the recently developed traditional and deep learning-based techniques for classifying lung nodules. Among these methods, CNN variants obtained an accuracy range of 83.4–99.6%, a specificity range of 73.3–95.17%, a sensitivity range of 73.3–96.85%, and an AUC range of 0.7755–0.9936, respectively. Several methods achieved high classification accuracy (>95%), including F&BGAN, Inception_ResNet_V2, ResNet152V2, ResNet152V2+GRU, CSO-CADLCC, ProCAN, Net121, ResNet50, DITNN, and optimal DBN with an opposition-based pity beetle algorithm. DCNN systems obtained a sensitivity of 89.3% [183] and an accuracy of 97.3% [184]. The classifier was developed based on the VGG19 and CNN models and achieved accuracy, sensitivity, specificity, recall, F1_score, AUC, and MCC above 98%. 

Forte et al. [209] recently conducted a systematic review and meta-analysis of the diagnostic accuracy of current deep learning approaches for lung cancer diagnosis. The pooled sensitivity and specificity of deep learning approaches for lung cancer detection were 93% and 68%, respectively. The results showed that AI plays an important role in medical imaging, but there are still many research challenges.

## 5. Challenges and Future Research Directions 

This study extensively surveys papers published between 2014 and 2022. Table 2, Table 3 and Table 4 demonstrate that deep learning-based lung imaging systems have achieved high efficiency and state-of-the-art performance for lung nodule segmentation, detection, and classification using existing medical images. Compared to reinforcement and supervised learning techniques, unsupervised deep learning techniques (such as CNN, Faster R-CNN, Mask R-CNN, and U-Net) are more popular methods that have been used to develop convolutional networks for lung cancer detection and false-positive reduction.

Previous studies have shown that CT is the most widely used imaging tool in the CAD system for lung cancer diagnosis. Compared to 2D CNN, 3D CNN architectures provide more promising usefulness in obtaining representative features of malignant nodules. To this day, only a few works on 3D CNN for lung cancer diagnosis have been reported. 

Deep learning techniques have achieved good performance in segmentation and classification. However, deep learning techniques still have many unsolved problems in lung cancer detection. First, clinicians have not fully acknowledged deep learning techniques for everyday clinical exercise due to the lack of standardized medical image acquisition protocols. The unification of the acquisition protocols could minimize it. 

Second, deep learning techniques usually require massive annotated medical images by experienced radiologists to complete training tasks. However, it is costly and time consuming to collect an enormous annotated image dataset, even performed by experienced radiologists. Several methods were applied to overcome the scarcity of annotated data. For example, transfer learning is a possible way to solve the training problem of small samples. Another possible method is the computer synthesis of images, such as the generation of confrontation networks. Inadequate data will inevitably affect the accuracy and stability of predictions. Therefore, improving prediction accuracy using weak supervision, transfer learning, and multi-task learning with small labeled data is one of the future research directions. 

Third, the clinical application of deep learning requires high interpretability, but current deep learning techniques cannot effectively explain the learned features. Many researchers have applied visualization and parameter analysis methods to explain deep learning models. However, there is still a certain distance from the interpretable imaging markers required by clinical requirements. Therefore, investigating the interpretable deep learning method will be a hot spot in the medical image field. 

Fourth, developing the robustness of the prediction model is a challenging task. Most deep learning techniques work well only for a single dataset. The image of the same disease may vary significantly due to different acquisition parameters, equipment, time, and other factors. This led to poor robustness and generalization of existing deep learning models. Thus, improving the model structure and training methods by combining brain cognitive ideas and improving the generalization ability of deep learning is one of the key future directions. 

Finally, some of the current literature has little translation into applicability in clinical practice due to the lack of experience of non-medical investigators in choosing more relevant clinical outcomes. Most deep learning techniques were developed by non-medical professionals with little or no oversight of radiologists, who, in practice, will use these resources when they become more widely available. As a result, some performance metrics, such as accuracy, AUC, and precision, which have little meaningful clinical application, continue to be used and are often the only summary outcomes reported by some studies. Instead, investigators should always strive to report more relevant clinical parameters, such as sensitivity and specificity, because they are independent of the prevalence of the disease and can be more easily translated into practice.

In the future, investigators should pay more attention to the following research directions: (1) develop new convolutional networks and loss functions to improve the performance; (2) weak supervised learning, using a large number of incomplete, inaccurate, and ambiguous annotation data in the existing medical records to achieve model training; (3) bring prior clinical knowledge into model training; (4) radiologists, computer scientists, and engineers need to work more closely to develop more realistic and sensitive models and add more meaning to the research field; (5) single disease identification to complete disease identification. In clinical examination, only a few cases need to solve one well-defined problem. For example, clinicians can detect pulmonary nodules in LDCT and check whether there are other abnormalities, such as emphysema. Solving multiple problems with one network will not reduce performance in specific tasks. In addition, deep learning can be explored in some areas where the medical mechanism is not precise, such as large-scale lung image analysis using deep learning, which is expected to make diagnosing lung diseases more objective.

## 6. Conclusions

This paper reviewed recent achievements in deep learning-based approaches for lung nodule segmentation, detection, and classification. CNN is one of the most widely used deep learning techniques for lung disease detection and classification, and CT image datasets are the most frequently used imaging datasets for training networks. The article review was based on recent publications (published in 2014 and later). Experimental and clinical trial results demonstrate that deep learning techniques can be superior to trained radiologists. Deep learning is expected to effectively improve lung nodule segmentation, detection, and classification. With this powerful tool, radiologists can interpret images more accurately. Deep learning algorithm has shown great potential in a series of tasks in the radiology department and has solved many medical problems. However, it still faces many difficulties, including large-scale clinical verification, patient privacy protection, and legal accountability. Despite these limitations, with the current trend and rapid development of the medical industry, deep learning is expected to generate a greater demand for accurate diagnosis and treatment in the medical field.

## Figures and Tables

**Table 1:** Lung image dataset.

Reference	Dataset	Sample Number
[53]	Lung image database consortium (LIDC)	399 CT images
[54]	Lung image database consortium and image database resource initiative (LIDC-IDRI)	1018 CT images from 1010 patients
[55]	Lung nodule analysis challenge 2016 (LUNA16)	888 CT images from LIDC-IDRI dataset
[56]	Early lung cancer action program (ELCAP)	50 LDCT lung images & 379 unduplicated lung nodule CT images
[57]	Lung Nodule Database (LNDb)	294 CT images from Centro Hospitalar e Universitario de São Joãao
[58]	Indian Lung CT Image Database (ILCID)	CT images from 400 patients
[59]	Japanese Society of Radiological Technology (JSRT)	154 nodules & 93 nonnodules with labels
[60]	Nederland-Leuvens Longkanker Screenings Onderzoek (NELSON)	CT images from 15,523 human subjects
[61]	Automatic nodule detection 2009 (ANODE09)	5 examples & 50 test images
[62]	Shanghai Zhongshan hospital database	CT images from 350 patients
[63]	Society of Photo-Optical Instrumentation Engineers in conjunction with the American Association of Physicists in Medicine and the National Cancer Institute (SPIE-AAPM-NCI) LungX	60 thoracic CT scans with 73 nodules
[64]	General Hospital of Guangzhou military command (GHGMC) dataset	180 benign & 120 malignant lung nodules
[65]	First Affiliated Hospital of Guangzhou Medical University (FAHGMU) dataset	142 T2-weighted MR images
[66]	Non-small cell lung cancer (NSCLC)-Radiomics database	13,482 CT images from 89 patients
[67]	Danish lung nodule screening trial (DLCST)	CT images from 4104 subjects
[68]	U.S. National Lung Screening Trial (NLST)	CT images from 1058 patients with lung cancer & 9310 patients with benign lung nodules

**Table 2:** Lung nodule segmentation approaches.

Reference	Year	Method	Imaging	Datasets	Results
[84]	2013	Support vector machine (SVM)	CT images	Shiraz University of Medical Sciences	Accuracy: 98.1%
[85]	2014	Lung nodule segmentation	CT images	85 patients	Accuracy: >90%
[86]	2015	SVM	CT images	193 CT images	Accuracy: 94.67% for benign tumors; Accuracy: 96.07% for adhesion tumor
[87]	2015	Bidirectional chain coding combined with SVM	CT images	LIDC	Accuracy: 92.6%
[88]	2015	Convolutional networks (ConvNets)	CT images	82 patients	DSC: 68% ± 10%
[77]	2017	Multi-view convolutional neural networks (MV-CNN)	CT images	LIDC-IDRI	DSC: 77.67%
[80]	2017	Two-stage CAD	CT images	LIDC-IDRI	F1-score: 85.01%
[89]	2017	3D Slicer chest imaging platform (CIP)	CT images	LIDC	median DSC: 99%
[90]	2017	Deep computer aided detection (CAD)	CT images	LIDC-IDRI	Sensitivity: 88%
[91]	2018	3D deep multi-task CNN	CT images	LUNA16	DSC: 91%
[92]	2018	Improved U-Net	CT images	LUNA16	DSC: 73.6%
[93]	2018	Incremental-multiple resolution residually connected network (MRRN)	CT images	TCIA	DSC: 74% ± 0.13
				MSKCC	DSC: 75%±0.12
				LIDC	DSC: 68%±0.23
[94]	2018	U-Net	hematoxylin-eosin-stained slides	712 lung cancer patients operated in Uppsala Hospital, Stanford TMA cores	Precision: 80%
[95]	2019	Mask R-CNN	CT images	LIDC-IDRI	Average precision:78%
[96]	2020	3D-UNet	CT images	LUNA16	DSC: 95.30%
[81]	2020	Dual-branch Residual Network (DB-ResNet)	CT images	LIDC-IDRI	DSC: 82.74%
[97]	2021	End-to-end deep learning	CT images	1916 lung tumors in 1504 patients	Sensitivity: 93.2%
[98]	2021	3D Attention U-Net	COVID-19 CT images	Fifth Medical Center of the PLA General Hospital	Accuracy: 94.43%
[99]	2021	Improved U-Net	CT images	LIDC-IDRI	Precision: 84.91%
[82]	2021	Attention-aware weight excitation U-Net (AWEU-Net)	CT images	LUNA16	DSC: 89.79%
				LIDC-IDRI	DSC: 90.35%
[83]	2021	Dense Recurrent Residual Convolutional Neural Network(Dense R2U CNN)	CT images	LUNA	Sensitivity: 99.4% ± 0.2%
[100]	2021	Modified U-Net in which the encoder is replaced with a pre-trained ResNet-34 network (Res BCDU-Net)	CT images	LIDC-IDRI	Accuracy: 97.58%
[101]	2021	Hybrid COVID-19 segmentation and recognition framework (HMB-HCF)	X-Ray images	COVID-19 dataset from 8 sources *	Accuracy: 99.30%
[102]	2021	Clinical image radionics DL (CIRDL)	CT Images	First Affiliated Hospital of Guangzhou Medical University	Sensitivity: 0.8763
[103]	2021	2D & 3D hybrid CNN	CT scans	260 patients with lung cancer treated	Median DSC: 0.73
[104]	2022	Few-shot learning U-Net (U-Net FSL)	PET/CT images	Lung-PET-CT-DX TCIA	Accuracy: 99.27% ± 0.03
		U-Net CT			Accuracy: 99.08% ± 0.05
		U-Net PET			Accuracy: 98.78% ± 0.06
		U-Net PET/CT			Accuracy: 98.92% ± 0.09
		CNN			Accuracy: 98.89% ± 0.08
		Co-learning			Accuracy: 99.94% ± 0.09
[105]	2022	DenseNet201	CT images	Seoul St. Mary’s Hospital dataset	Sensitivity: 96.2%

COVID-19 dataset from 8 sources *: COVID-19 Radiography Database, Pneumonia (virus) vs. COVID-19 Dataset, Covid-19 X-Ray images using CNN Dataset, COVID-19 X-ray Images5 Dataset, COVID-19 Patients Lungs X-Ray Images 10,000 Dataset, COVID-19 Chest X-Ray Dataset, COVID-19 Dataset, Curated Chest X-Ray Image Dataset for COVID-19.

**Table 3:** Lung nodule detection approaches.

Reference	Year	Method	Imaging	Datasets	Results
[122]	2016	3D CNN	CT images	LUNA16	Sensitivity: >87% at 4 FPs/scan
[123]	2016	2D multi-view convolutional networks (ConvNets)	CT images	LIDC-IDRI	Sensitivity: 85.4% at 1 FPs/scan, 90.1% at 4 FPs/scan
[124]	2016	Thresholding method	CT images	JSRT	Accuracy: 96%
[110]	2017	Computer aided detection (CAD)	LDCT	NLST	Mean sensitivity: 74.1%
[125]	2017	3D CNN	LDCT	KDSB17	Accuracy: 87.5%
[126]	2017	3D Faster R-CNN with U-Net-like encoder	CT scans	LUNA16	Accuracy: 81.41%;
				LIDC-IDRI	Accuracy: 90.44%
[127]	2018	Single-view 2D CNN	CT scans	LUNA16	metric score: 92.2%
[128]	2018	DetectNet	CT scans	LIDC	Sensitivity: 89%
[129]	2018	3D CNN	CT scans	KDSB17	Sensitivity: 87%;
[130]	2018	Novel pulmonary nodule detection algorithm (NODULe) based on 3D CNN	CT scans	LUNA16	CPM score: 94.7%
				LIDC-IDRI	Sensitivity: 94.9%
[131]	2018	Deep neural networks (DNN)	PET images	50 lung cancer patients, & 50 patients without lung cancer	Sensitivity: 95.9%
			ultralow dose PET		Sensitivity: 91.5%
[132]	2018	FissureNet	3DCT	COPDGene	AUC: 0.98
		U-Net			AUC: 0.963
		Hessian			AUC: 0.158
[133]	2018	DFCN-based cosegmentation (DFCN-CoSeg)	CT scans	60 NSCLC patients	Score: 0.865 ± 0.034;
			PET images		Score: 0.853 ± 0.063;
[134]	2018	Multi-scale Gradual Integration CNN (MGI-CNN)	CT scans	LUNA16, V1 dataset includes 551,065 subjects; V2 dataset includes 754,975 subjects	CPM: 0.908 for the V1 dataset, 0.942 for the V2 dataset;
[135]	2018	Deep fully CNN (DFCNet)	CT scans	LIDC-IDR	Accuracy: 84.58%
		CNN			Accuracy: 77.6%
[136]	2018	Deep learning–based automatic detection algorithm (DLAD)	CT scans	Seoul National University Hospital	Sensitivity: 69.9%
[137]	2018	SVM classifier coupled with a least absolute shrinkage and selection operator (SVM-LASSO)	CT scans	LIDC-IDRI	Accuracy: 84.6%
[138]	2019	CNN	CT scans	LIDC-IDR	Sensitivity: 88% at 1.9 FPs/scan; 94.01% at 4.01 FPs/scan
[139]	2019	3D CNN	LDCT	LUNA16 and Kaggle datasets	Average metric: 92.1%
[140]	2019	Deep learning model (DLM) based on DCNN	Chest radiographs (CXRs)	3500 CXRs contain lung nodules & 13,711 normal CXRs	Sensitivity: 76.8%
[141]	2019	Two-Step Deep Learning	CT scans	Nagasaki University Hospital	Sensitivity of 79.6% with sizes ≤0.6 mm; Sensitivity of 75.5% with sizes ≤0.7 mm;
[142]	2019	Faster R-CNN network and false positive (FP)	CT scans	FAHGMU	Sensitivity: 85.2%
[143]	2019	YOLOv2 with Asymmetric Convolution Kernel	CT scans	LIDC-IDRI	Sensitivity: 94.25%
[144]	2019	VGG-16 network	CT scans	LIDC-IDRI	Accuracy: 92.72%
[145]	2019	Noisy U-Net (NU-Net)	CT scans	LUNA16	Sensitivity: 97.1%
[146]	2019	CAD using a multi-scale dot nodule-enhancement filter	CT scans	LIDC	Sensitivity: 87.81%
[147]	2019	Co-Learning Feature Fusion CNN	PET-CT scans	50 NSCLC patients	Accuracy: 99.29%
[148]	2019	Convolution networks with attention feedback (CONAF)	Chest radiographs	430,000 CXRs	Sensitivity: 78%
[148]	2019	Recurrent attention model with annotation feedback (RAMAF)	Chest radiographs	430,000 CXRs	Sensitivity: 74%
[113]	2020	Two-Stage CNN (TSCNN)	CT scans	LUNA16 & LIDC-IDRI	CPM: 0.911
[149]	2020	Deep Transfer CNN and Extreme Learning Machine (DTCNN-ELM)	CT scans	LIDC-IDRI & FAH-GMU	Sensitivity: 93.69%;
[150]	2020	U-Net++	CT scans	LIDC-IDRI	Sensitivity: 94.2% at 1 FP/scan, 96% at 2 FPs/scan
[151]	2020	MSCS-DeepLN	CT scans	LIDC-IDRI & DeepLN	
[152]	2020	Multi-scale CNN (MCNN)	CT scans	LIDC-IDRI	Accuracy: 93.7% ± 0.3
[153]	2021	Lung Cancer Prediction CNN (LCP-CNN)	CT scans	U.S. NLST	Sensitivity: 99%;
[154]	2021	Automatic AI-powered CAD	CT scans	150 images include 340 nodules	mean sensitivity: 82% for second-reading mode, 80% for concurrent-reading mode
[155]	2021	DNA-derived phage nose (D2pNose) using machine learning and ANN	CT scans	Pusan National University	Detection accuracy: >75%; Classification accuracy: >86%
[156]	2021	Capsule network-based mixture of experts (MIXCAPS)	CT scans	LIDC-IDRI	Sensitivity: 89.5%;
[157]	2021	CNN with attention mechanism	CT scans	LUNA16	Specificity: 98.9%
[121]	2021	Deep learning image reconstruction (DLIR)	CT scans	LIDC-IDRI	AUC of 0.555, 0.561, 0.557, 0.558 for ASIR-V, DL-L, DL-M, DL-H
[58]	2021	2D-3D cascaded CNN	CT scans	LIDC-IDRI	Sensitivity: 90.01%
[120]	2022	3D sphere representation-based center-points matching detection network (SCPM-Net)	CT scans	LUNA16	Average sensitivity: 89.2%
[158]	2022	YOLOv3	CT scans	RIDER	Accuracy: 95.17%
[118]	2022	3D Attention CNN	CT scans	LUNA16	CPM: 0.931
[159]	2022	Progressive Growing Channel Attentive Non-Local (ProCAN) network	CT scans	LIDC-IDRI	Accuracy: 95.28%

**Table 4:** Lung nodule classification approaches.

Reference	Year	Method	Imaging	Datasets	Results
[185]	2014	FF-BPNN	CT scans	LIDC	Sensitivity: 91.4%
[168]	2015	Multi-scale CNN	CT scans	LIDC-IDRI	Accuracy: 86.84%
[166]	2015	CAD using deep features	CT scans	LIDC-IDRI	Sensitivity: 83.35%
[165]	2015	Deep belief network (DBN)	CT scans	LIDC	Sensitivity: 73.4%
[165]	2015	CNN	CT scans	LIDC	Sensitivity:73.3%
[165]	2015	Fractal	CT scans	LIDC	Sensitivity:50.2%
[165]	2015	Scale-invariant feature transform (SIFT)	CT scans	LIDC	Sensitivity: 75.6%
[186]	2016	Intensity features +SVM	CT scans	DLCST	Accuracy: 27.0%
[186]	2016	Unsupervised features+SVM	CT scans	DLCST	Accuracy: 39.9%
[186]	2016	ConvNets 1 scale	CT scans	DLCST	Accuracy: 84.4%
[186]	2016	ConvNets 2 scale	CT scans	DLCST	Accuracy: 85.6%
[186]	2016	ConvNets 3 scale	CT scans	DLCST	Accuracy: 85.6%
[171]	2017	Multi-crop CNN	CT scans	LIDC-IDRI	Accuracy: 87.14%
[171]	2017	Deep 3D DPN	CT scans	LIDC-IDRI	Accuracy: 88.74%
[171]	2017	Deep 3D DPN+ GBM	CT scans	LIDC-IDRI	Accuracy: 90.44%
[111]	2017	Massive-training ANN (MTANN)	CT scans	LDCT	AUC: 0. 8806
[111]	2017	CNN	CT scans	LDCT	AUC: 0.7755
[187]	2017	Wavelet Recurrent Neural Network	Chest X-Ray	Japanese Society Radiology and Technology	Sensitivity: 88.24%
[171]	2017	Multi-crop convolutional neural network (MC-CNN)	CT scans	LIDC-IDRI	Sensitivity: 77%
[188]	2018	Topology-based phylogenetic diversity index classification CNN	CT scans	LIDC	Sensitivity: 90.70%
[189]	2018	Transfer learning deep 3D CNN	CT scans	Institution records	Accuracy: 71%
[128]	2018	CNN	CT scans	Kaggle Data Science Bowl 2017	Sensitivity: 87%
[190]	2018	Feature Representation Using Deep Autoencoder	CT scans	ELCAP	Accuracy: 93.9%
[112]	2018	Multi-view multi-scale CNN	CT scans	LIDC-IDRI & ELCAP	overall classification rates: 92.3% for LIDC-IDRI; overall classification rates: 90.3% for ELCAP
[191]	2018	Wavelet-Based CNN	CT scans	448 images include four categories	Accuracy: 91.9%
[192]	2018	Deep ConvNets	CT scans	LIDC-IDRI	Accuracy: 98%
[182]	2018	Forward and Backward GAN (F&BGAN)	CT scans	LIDC-IDRI	Sensitivity: 98.67%
[174]	2019	Ensemble learner of multiple deep CNN	CT scans	LIDC-IDRI	Accuracy: 84.0%
[175]	2019	Lightweight Multi-Section CNN	CT scans	LIDC-IDRI	Accuracy: 93.18%
[167]	2019	Deep hierarchical semantic CNN (HSCNN)	CT scans	LIDC	Sensitivity: 70.5%
[193]	2019	Multi-view knowledge-based collaborative (MV-KBC)	CT scans	LIDC-IDRI	Accuracy: 91.60%
[167]	2019	3D CNN	CT scans	LIDC	Sensitivity: 66.8%
[183]	2019	DCNN	CT scans	46 images from interventional cytology	Sensitivity: 89.3%
[194]	2019	3D MixNet	CT scans	LIDC-IDRI & LUNA16	Accuracy: 88.83%
[194]	2019	3D MixNet +GBM	CT scans	LIDC-IDRI & LUNA16	Accuracy: 90.57%
[194]	2019	3D CMixNet+ GBM	CT scans	LIDC-IDRI & LUNA16	Accuracy: 91.13
[194]	2019	3D CMixNet+ GBM+Biomarkers	CT scans	LIDC-IDRI & LUNA16	Accuracy: 94.17%
[195]	2019	Deep Learning with Instantaneously Trained Neural Networks (DITNN)	CT scans	Cancer imaging Archive (CIA)	Accuracy: 98.42%
[184]	2020	DCNN	CT scans	LIDC	Accuracy: 97.3%
[196]	2020	CNN	CT scans	LIDC	Sensitivity: 93.4%
[197]	2020	2.75D CNN	CT scans	LUNA16	AUC: 0.9842
[198]	2020	Two-step Deep Network (TsDN)	CT scans	LIDC-IDRI	Sensitivity: 88.5%
[176]	2020	Transferable texture CNN	CT scans	LIDC-IDRI & LUNGx	Accuracy: 96.69% ± 0.72%
[199]	2020	Taguchi-Based CNN	X-ray & CT images	245,931 images	Accuracy: 99.6%
[200]	2021	Optimal Deep Belief Network with Opposition-based Pity Beetle Algorithm	CT scans	LIDC-IDRI	Sensitivity: 96.86%
[177]	2021	Multi-task CNN	CT scans	LIDC-IDRI	AUC: 0.783
[178]	2021	CNN	CT scans	JSRT	Accuracy: 86.67%
[201]	2021	Inception_ResNet_V2	CT scans	LC25000	Accuracy: 99.7%
[201]	2021	VGG19	CT scans	LC25000	Accuracy: 92%
[201]	2021	ResNet50	CT scans	LC25000	Accuracy: 99%
[201]	2021	Net121	CT scans	LC25000	Accuracy: 99.4%
[202]	2021	Improved Faster R-CNN and transfer learning	CT scans	Heilongjiang Provincial Hospital	Accuracy: 89.7%
[203]	2021	Three-stream network	CT scans	LIDC-IDRI	Accuracy: 98.2%
[204]	2021	FractalNet	CT scans	LUNA 16	Sensitivity: 96.68%
[205]	2021	VGG19+CNN	X-ray & CT images	GitHub	Specificity: 99.5%
[205]	2021	ResNet152V2	X-ray & CT images	GitHub	Specificity: 98.4%
[205]	2021	ResNet152V2+GRU	X-ray & CT images	GitHub	Specificity: 98.7%
[205]	2021	ResNet152V2+Bi-GRU	X-ray & CT images	GitHub	Specificity: 97.8%
[179]	2022	Machine learning	CT scans	LNDb	Accuracy: 94%
[159]	2022	Progressively Growing Channel Attentive Non-Local (ProCAN)	CT scans	LIDC-IDRI	Accuracy: 95.28%
[206]	2022	CNN-based multi-task learning (CNN-MTL)	CT scans	LIDC-IDRI	Sensitivity: 96.2%
[207]	2022	Cat swarm optimization-based CAD for lung cancer classification (CSO-CADLCC)	CT scans	Benchmark	Specificity: 99.17%
[208]	2022	2-Pathway Morphology-based CNN (2PMorphCNN)	CT scans	LIDC-IDRI	Sensitivity: 96.85%

## Data Availability

Not applicable.
